# SOX on tumors, a comfort or a constraint?

**DOI:** 10.1038/s41420-024-01834-6

**Published:** 2024-02-09

**Authors:** Junqing Jiang, Yufei Wang, Mengyu Sun, Xiangyuan Luo, Zerui Zhang, Yijun Wang, Siwen Li, Dian Hu, Jiaqian Zhang, Zhangfan Wu, Xiaoping Chen, Bixiang Zhang, Xiao Xu, Shuai Wang, Shengjun Xu, Wenjie Huang, Limin Xia

**Affiliations:** 1 Department of Gastroenterology, Institute of Liver and Gastrointestinal Diseases, Hubei Key Laboratory of Hepato-Pancreato-Biliary Diseases, Tongji Hospital of Tongji Medical College, Huazhong University of Science and Technology, Wuhan, 430030 Hubei Province China; 2 Hubei Key Laboratory of Hepato-Pancreato-Biliary Diseases; Hepatic Surgery Center, Tongji Hospital, Tongji Medical College, Huazhong University of Science and Technology; Clinical Medicine Research Center for Hepatic Surgery of Hubei Province; Key Laboratory of Organ Transplantation, Ministry of Education and Ministry of Public Health, Wuhan, Hubei 430030 China; 3 Key Laboratory of Integrated Oncology and Intelligent Medicine of Zhejiang Province, Department of Hepatobiliary and Pancreatic Surgery, Affiliated Hangzhou First People’s Hospital, Zhejiang University School of Medicine, Hangzhou, 310006 China; 4 Key Laboratory of Integrated Oncology and Intelligent Medicine of Zhejiang Province, Department of Hepatobiliary and Pancreatic Surgery, Affiliated Hangzhou First People’s Hospital, Westlake university school of medicine, Hangzhou, 310006 China

**Keywords:** Cancer genomics, Cancer immunotherapy

## Abstract

The sex-determining region Y (SRY)-related high-mobility group (HMG) box (SOX) family, composed of 20 transcription factors, is a conserved family with a highly homologous HMG domain. Due to their crucial role in determining cell fate, the dysregulation of SOX family members is closely associated with tumorigenesis, including tumor invasion, metastasis, proliferation, apoptosis, epithelial-mesenchymal transition, stemness and drug resistance. Despite considerable research to investigate the mechanisms and functions of the SOX family, confusion remains regarding aspects such as the role of the SOX family in tumor immune microenvironment (TIME) and contradictory impacts the SOX family exerts on tumors. This review summarizes the physiological function of the SOX family and their multiple roles in tumors, with a focus on the relationship between the SOX family and TIME, aiming to propose their potential role in cancer and promising methods for treatment.

## Facts

- Acetylation and SUMOylation promotes the translocation of SOX TFs from nucleus to cytoplasm.
- SOX is overexpressed in multiple tumors and promotes tumor occurrence and development by promoting EMT, stemness, metastasis, and proliferation.
- The SOX family promotes the formation of an inhibitory tumor immune microenvironment by regulating the differentiation activation of immune cells and the release of chemokines.

## Open questions

- Does the subcellular localization of SOX family differ in the occurrence and development of tumors? Is inhibiting or promoting this displacement effective for tumor treatment?
- What is the reason for the contradictory role that the SOX family may play in the same downstream signaling pathway in different or even the same tumors?
- The vast majority of research has focused on the three members of SOX2, SOX4, and SOX9. Do other members, particularly those in the same subgroup also play important roles in tumors?
- Besides serving as a tumor specific antigen, can the regulatory effect of SOX in immune microenvironment be utilized to develop a wider range of effective immunotherapy drugs?

## Introduction

Transcription factors (TFs) regulate gene expression by binding to upstream sequences or distal gene elements of the transcription start site. This regulation of gene expression is crucial in the processes of cell proliferation and differentiation [1]. Of all protooncogenes, approximately 19% are transcription factors [2]. Their mutations and aberrant activation exert a significant impact on tumor development and provide potential targets for therapies.

The SOX family was first discovered in mice in 1990, and since then its function has been gradually uncovered [3]. The SOX family plays an essential role in the development of many tissues and organs. Additionally, its involvement in sex determination, maintenance of stem cell function, and differentiation of immune cells has been gradually revealed [4]. Dysfunction of the SOX family leads to the manifestation of various diseases, the most serious of which is cancer.

In this review, we first discuss the structure and function of SOX TFs. Next, we reveal their multiple roles in tumor invasion and metastasis, proliferation and apoptosis, stemness and epithelial-mesenchymal transition (EMT). Finally, we highlight the involvement of the SOX family in the tumor immune microenvironment (TIME) and propose potential therapeutic targets and strategies to address drug resistance.

## General overview of SOX TFs

### Structures of SOX TFs

**Fig. 1:**
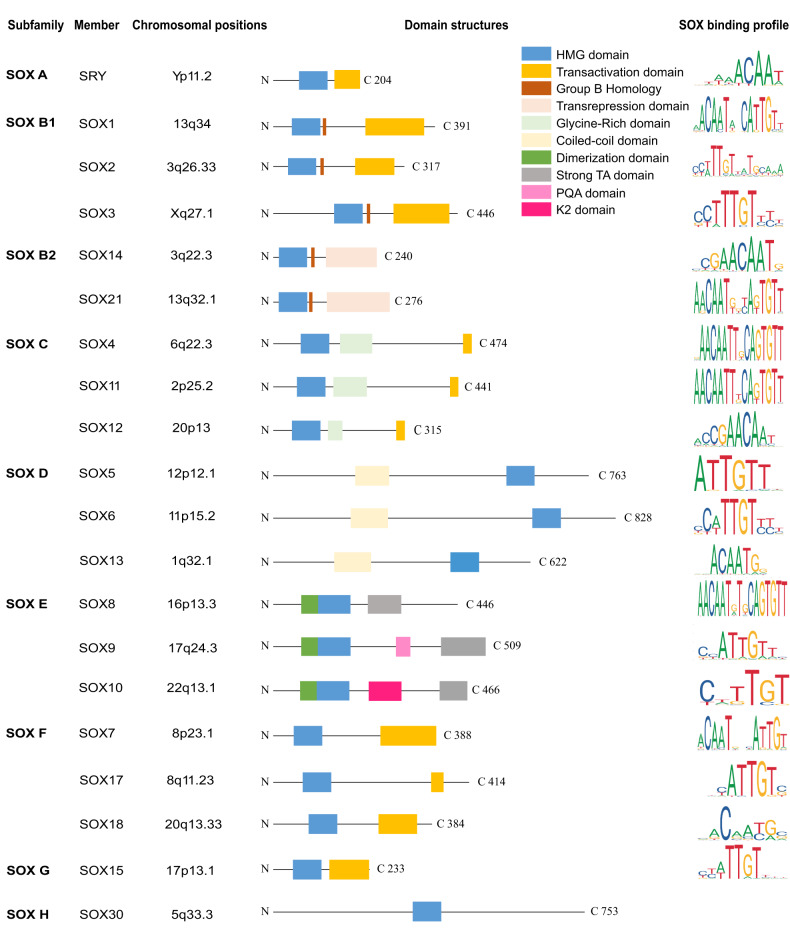
Domain structure and binding profile of human SOX transcription factors. The SOX family comprises nine groups, each exhibiting a highly conserved HMG domain spanning 79 amino acids. Members within the same subgroup exhibit similar structure and functionality. The diagram illustrates several domains, including the high mobility group (HMG) domain (blue box), homology domain of SOXB (red box), activation domain (orange box), inhibitory domain (pink box), dimerization domain (dark green box), glycine-rich domain (light green), strong TA domain (gray box). The chromosomal positions and domain structures of SOX members have been confirmed. The predicted SOX binding profiles were obtained from the JASPAR public database.

The sex-determining region Y (SRY)-related high-mobility group (HMG) box (SOX) family was first identified due to the homology between its HMG domain and the testicular determining factor SRY [3]. SOX TFs are a conserved family with highly homologous HMG domain, which mediate DNA binding and regulate gene expression [5]. The core domain of the SOX TFs, HMG, contains 79 amino acids residues with a hexameric core sequence WWCAAW (W = A/T) [4]. The sequence RPMNAFMVW is conserved in all SOX TFs except SRY [5]. To date, 20 members of the SOX TFs have been identified in mammals. According to the similarity of their HMG domain, SOX TFs are classified into 8 different subgroups: subgroup A (SRY), subgroup B1 (SOX1, SOX2, SOX3), subgroup B2 (SOX14, SOX21), subgroup C (SOX4, SOX11, SOX12), subgroup D (SOX5, SOX6, SOX13), subgroup E (SOX8, SOX9, SOX10), subgroup F (SOX7, SOX17, SOX18), SOX G (SOX15), SOX H (SOX30) (Fig. 1). Among the subgroups, members share similar functions based on their similar structure. For example, SOX4 and SOX11 in subgroup C possess similar glycine-rich areas in the middle of their domain and exhibit synergistic effects on the neurogenesis [6] and inducing the formation of cartilage growth plate [7].

### Physiological function of SOX TFs

SOX TFs are crucial for numerous aspects of organizational development, including the cardiovascular, skeletal, pulmonary and nervous systems. Besides, they also participate in sex differentiation and stem cell homeostasis maintenance.

#### Blood cell

The SOX family maintains the stemness of hematopoietic stem cells and regulates their differentiation and maturation. SOX4 facilitates T lymphocyte differentiation in the thymus [8], while SOX6 supports the survival and maturation of erythroid cells [9]. SOX7 regulates the mesodermal bloodline and promotes the formation of hematopoietic progenitor cells and endothelial progenitor cells [10]. SOX17 primes hemogenic potential in endothelial cells (ECs), thereby regulating hematopoietic development from human embryonic stem cells (hESCs)/ induced pluripotent stem cells (iPSCs) [11].

#### Neuron

The SOX family promotes the development of the central nervous system and peripheral nerves, as well as facilitates the regeneration and repair of damaged nerves. SOX2 promotes the relocalization of N-cadherin to Schwann cells, promoting axonal regrowth [12]. Members in SOX B1 maintain stem cells in CNS, while SOX10 promotes terminal oligodendrocyte differentiation [13]. SOX9 is essential in the formation and maintenance of multipoint neural stem cells [14]. SOX10 is a transcriptional regulator of Schwann cell differentiation and maturation, promoting peripheral nervous system development [15]. The premature termination of SOX10 leads to absence of neural crest in Hirschsprung disease [16].

#### Cardiovascular

By regulating the proliferation and differentiation of myocardial cells and vascular endothelial cells, the SOX family promotes cardiovascular development. SOX4^-/-embryos^ stuck into the endothelial ridges without further development into the semilunar valves and the outlet portion of the muscular ventricular septum [17]. SOX6 participates in the regulation of L-type Ca2+channels, regulating cardiac myocyte development [18]. SOX7, as the first direct endothelial-specific regulator of Vegfc transcription, can strictly control the number and spatial distribution of lymphatic endothelial cells (LECs) by locally suppressing VEGFC levels [19]. SOX9 is essential for the formation of cardiac valves and septa, while the loss of SOX9 fails to form the endocardial cushions [20]. SOX17 participates in cardiovascular development, helping to form the cardiac muscle [21]. Besides, SOX17 promotes coronary vessels development via activating Nestin’s enhancer [22]. SOX18 and SOX7 has a redundant role in the establishment of proper arteriovenous identity in zebrafish [23].

#### Sex determination

The SOX family plays a crucial role in the development of male reproductive organs and gender determination. SRY, the only Y-linked gene required to give rise to male development, is able to induce sex reversal in XX transgeneic adult mice [24]. Similarly, SOX9 fosters the development of double potential gonads into testes, which is integral to testicular development [25]. The mutations of SOX9 leads to autosomal sex reversal and campomelic dysplasia [26]. Besides, SOX8 and SOX9 have redundancy in Sertoli cell development, cord formation and testis differentiation. Clarkson et al. believed that such redundancy, which is typical feature of SOX TFs biology, represents a mere evolutionary relic in mammals [27].

#### Lung development

The SOX family regulates alveolar and bronchial differentiation, promotes the formation of pulmonary vasculature, and contributes to pulmonary tissue development. They are closely associated with pulmonary arterial hypertension. SOX2, SOX9, and SOX17 exhibit a synergistic effect on the occurrence and maintenance of lung tissue. SOX2 is involved in the development of the bronchial branches, primarily expressed in the epithelium of the embryonic proximal airways, while hardly expressed in the adult lungs [28]. SOX2/SOX9 allows the manipulation of branching morphogenesis and the later formation of conducting and respiratory airways [29]. SOX9 promotes the proliferation of lung progenitor cells and inhibits premature initiation of airway and alveolar differentiation [30, 31]. By controlling the balance of proliferation and differentiation, and regulating extracellular matrix (ECM), SOX9 facilitates appropriate morphogenesis of branching [32]. Utilizing a rare population of SOX9 basal cells (BCs) located at airway epithelium rugae, Ma et al. regenerate adult human lung [33]. SOX17 promotes the differentiation of precursor cells into alveolar epithelial cells and bronchial epithelial cells, and regulates the expression of their specific genes [34]. Besides, SOX17 promotes the differentiation of endothelial cells and vascular formation, the variation of its enhancers is one of the important causes for the formation of pulmonary arterial hypertension [35].

#### Cartilage

Through regulating ECM formation and cellular metabolism, the SOX family participates in cartilage formation and regeneration [36]. As an essential regulator of skeletal progenitor cells, SOX9 plays an important role in cartilage formation, cartilage regeneration, and endochondral osteogenesis [36, 37]. Besides, SOX5, SOX6, and SOX9 are often referred to as SOX Trio, which together regulate the development of cartilage. Even with normal expression of SOX9, knocking out SOX5 and SOX6 simultaneously can lead to mouse death in the uterus due to chondrodysplasia [38]. Through regulating ECM formation and cellular metabolism, the SOX family plays a vital role in the development and regeneration of cartilage [36].

In summary, SOX plays an important regulatory role in different stages of organ growth and development. This requires precise spatiotemporal regulation. Precise regulation of SOX gene expression occurs at multiple levels, including transcriptional and translational regulation, as well as post-translational modification (PTM) [39]. These regulatory systems collectively maintain the normal physiological functions of the SOX family within the human body and, when anomalies occur, can lead to numerous diseases, cancer included (Table 1).

**Table 1:** The physical function of SOX family and their role in cancer.

Subfamily	Member	Physiological function	Role in cancer	Reference
SOX A	SRY	Sex determination	Promotes the migration and invasion of HCC cells	[24, 185, 186]
SOX B1	SOX1	Neural stem cell differentiation	Inhibits the invasion of HCC, lung cancer, nasopharyngeal carcinoma	[132, 187]
	SOX2	Maintains embryonic stem cell properties Axonal regrowth Occurrence and maintenance of lung tissue.	Inhibits invasion of ESCC, GC Promotes metastasis of bladder cancer, GBM, CRC, lung cancer	[12, 67, 94, 103–105, 137, 138, 188, 189]
	SOX3	Indispensable for gonadal function	Promotes migration and metastasis of GC and osteosarcoma	[190]
SOX B2	SOX14	Promotes neurogenesis	Participates in the proliferation and invasion of cervical cancer	[191]
	SOX21	Neural stem cell differentiation	Induces apoptosis of GBM and glioma	[127, 192]
SOX C	SOX4	Lymphocyte differentiation Cartilage growth plate formation, neurogenesis, cardiovascular development	Promotes proliferation and metastasis of ESCC, TNBC, CRC, OSCC, HCC	[6, 7, 17, 54, 58, 68, 95, 99]
	SOX11	Neurogenesis Formation of cartilage growth plate	Inhibits the proliferation of hematopoietic malignancies and Ewing sarcoma Induces EMT in uterine carcinosarcoma	[6, 7, 59, 86, 129, 193]
	SOX12	Lymphocyte differentiation	Increases proliferation and metastasis of HCC and GC	[91, 96, 143]
SOX D	SOX5	Interference of oligodendrocyte specification Activation of neural crest specification Cartilage formation	Induces apoptosis of glioma Promotes metastasis of NSCLC, prostate cancer, CRC	[38, 81, 127, 194]
	SOX6	Survival and maturation of erythroid cells Development of cardiac myocyte and cartilage	Inhibits proliferation and metastasis of glioma, pancreatic cancer, HCC Promotes EMT in NPC	[9, 18, 38, 56, 126, 127]
	SOX13	Lymphocyte differentiation	Promotes metastasis of HCC, CRC Promotes proliferation of BC, thyroid cancer	[85, 98, 141]
SOX E	SOX8	Involved in testis differentiation, osteogenesis,	Promotes proliferation of proliferation of gestational trophoblastic neoplasia and TSCC	[24, 195, 196]
	SOX9	Development of testicular and lung tissue, maintains the homeostasis of stem cells in the tissue Promotes delamination of the trunk neural crest	Tumor suppressor in melanoma Promotes metastasis of HCC, CRC, prostate cancer Promotes proliferation of HCC, CRC, GC	[25, 70, 71, 89, 90, 108–112, 163, 193, 197]
	SOX10	Peripheral nervous development	Promotes EMT in HCC, biomarker for breast cancer	[84, 171]
SOX F	SOX7	Hematopoietic development, regulates lymphatic endothelial cells	Inhibits proliferation of lung cancer, CRC and AML, Treg infiltration in glioma, recruitment of TAMs	[19, 123, 124, 128, 150, 154, 198]
	SOX17	Development of lung tissue and cardiovascular	Promotes angiogenesis in ovarian cancer, recruit Tregs in glioma	[21, 100, 193]
	SOX18	Development of the endothelial cells lining the blood and lymphatic vessels heart and skeletal muscle	Induces metastasis of GC cells	[23, 93, 199]
SOX G	SOX15	Population of myogenic progenitor cells and regeneration	Inhibits proliferation of prostate cancer and glioma	[125, 130]
SOX H	SOX30	Promotes or maintain meiosis and gender differentiation of testicular germ cell	Inhibits invasion and metastasis of lung cancer and prostate cancer	[64, 200]

*AML* acute myeloid leukemia, *BC* breast cancer, *CRC* colorectal cancer, *EMT* epithelial-mesenchymal transition, *ESCC* esophageal squamous cell carcinoma, *GBM* glioblastoma, *GC* gastric cancer, *HCC* hepatic cell carcinoma, *NSCLC* non-small cell lung cancer, *NPC* nasopharyngeal carcinoma, *OSCC* oral squamous cell carcinoma, *SOX* sex determining region Y (SRY)- HMG box, *TAM* tumor-associated macrophages, *TNBC* triple-negative breast cancer, *TREG* regulatory T cell, *TSCC* tongue squamous cell carcinoma.

## Regulation of SOX TFs

### Post-translational modifications of SOX TFs

**Fig. 2:**
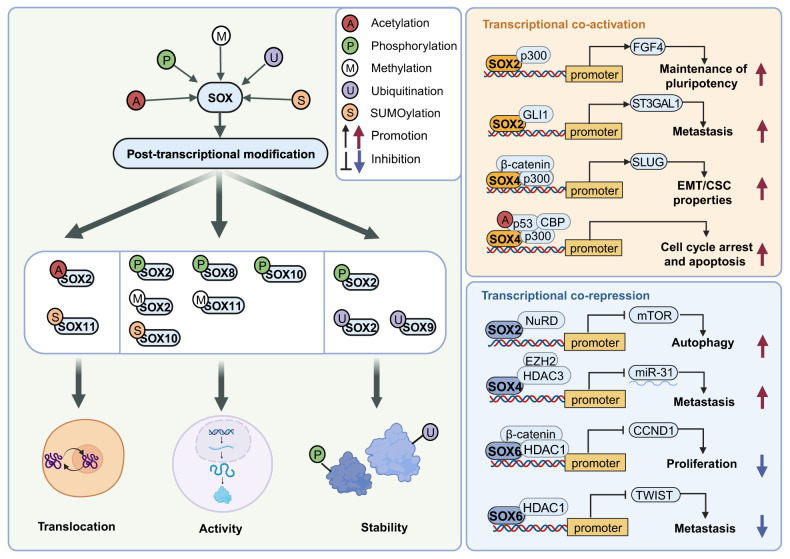
Post-transcriptional modifications and cofactors of SOX TFs. Post-transcriptional modification of SOX TFs mainly includes acetylation [48], phosphorylation [41, 42, 120], methylation [40, 59], ubiquitination [45, 46] and SUMOylation [43, 44], which infects the translocation, activity and stability of SOX TFs. Cofactors interact with SOX TFs and exert co-activation [50–53] or co-repression [54–57] effects on the physiological and pathological functions of SOX TFs.

PTMs serve as crucial regulatory mechanisms for modulating the expression of SOX TFs and orchestrating various oncogenic processes by controlling their localization, activity, and interactions with partner molecules. Elucidating intricate mechanisms of PTMs in the SOX family is of immense importance in advancing novel therapeutic targets. PTM refers to covalent modifications of proteins after RNA translation, mainly involving the addition of chemical groups to one or multiple residues of the target protein or small molecules to form the protein [40]. As for SOX TFs, most studies regarding PTMs concentrate on SOX2, SOX4 and SOX9. Here, the phosphorylation, SUMOylation, methylation, acetylation and ubiquitination of SOX TFs are presented (Fig. 2).

Phosphorylation, the most prevalent form of PTM, is critical for regulating protein activity and function [40]. PKCι phosphorylates Thr118 of SOX2, which upregulates hedgehog acyltransferase (HHAT) and maintains the stem-like and tumorigenic properties of lung squamous cell carcinoma (LSCC) [41]. Moreover, in melanoma, the phosphorylation of SOX10 by ERK at T240 and T244 residues inhibits SOX10 SUMOylation, dampening its transcriptional activity [42]. SUMOylation mainly involves the reversible binding of Small Ubiquitin-like Modifier (SUMO) to the target protein, which usually reduces DNA binding activity [40]. In nasopharyngeal carcinoma (NPC), phosphorylation of SOX2 by ERK permits its subsequent SUMOylation at K245, leading to the autophagic degradation of SOX2 [43]. SUMOylation of SOX11 suppresses its nuclear localization and binding ability. This mechanism is correlated with the proper expression of SOX4, which is critical for retinal ganglion cell development [44]. Ubiquitination often affects protein stability and promotes its degradation [40]. Setd7 mediates the methylation of SOX2 at K119, resulting in the ubiquitination and subsequent degradation of SOX2 [45]. Moreover, DDRGK domain-containing protein 1 (DDRGK1) inhibits ubiquitination and the subsequent degradation of SOX9 during chondrogenesis [46]. Methylation and acetylation are another two important forms of PTM. Methylation mainly impacts pioneer factor activity, while acetylation of SOX TFs promotes nuclear localization [40]. In embryonic stem cells (ESCs), methylation of SOX2 by CARM1 at Arg113 promotes SOX2 self-association and enhances SOX2 transactivation [47]. Regarding acetylation, p300 and CEP promote the acetylation of SOX2, while the mSin3A/ histone deacetylases (HDAC) complex promotes its deacetylation. The interplay between these proteins acts as a switch, rapidly regulating gene expression and facilitating nuclear export [48].

In addition to these chemical modifications, transcription factors can achieve co-activation or co-repression through protein-protein interactions. P300 and its homolog, CBP, are typical co-activators that serve as bridging molecules connecting promoters and proximal enhancer regions [49]. SOX2 interacts with p300 and cooperatively activates FGF4, maintaining pluripotency in embryonal carcinoma [50]. In uterine carcinosarcoma, the complex comprising SOX4, β-catenin, and p300 promotes β-catenin-mediated transcription of SLUG, contributing to EMT/CSC properties [51]. DNA damage response (DDR) acts as a tumorigenesis barrier. In DDR-associated cancer, Pan et al. discovered that SOX4 enhances p53 acetylation via interacting with p300/CBP and facilitating p300/CBP/p53 complex formation, which further promotes cell cycle arrest and apoptosis [52]. Besides p300, in melanoma, SOX2 forms a complex with GLI1 and cooperatively transactivates ST3GAL1, which promotes melanoma metastasis via upregulating AXL [53]. Similarly, HDAC and NuRD are the main co-repressors of SOX TFs. HDAC is a typical co-repressor factor that often exerts repressive functions through increasing the chromatin compaction of their target genes [40]. In esophageal squamous cell carcinoma (ESCC), SOX4 cooperates with EZH2 and HDAC3 to enhance tumor cell progression and metastasis through epigenetic silencing of miR-31 by H3K27me [54]. Through interacting with β-catenin and HDAC1, SOX6 suppresses the activity of the cyclin D1 (CCND1) promoter, while downregulation of SOX6 induces the proliferation of pancreatic β-cells [55]. In pancreatic cancer, SOX6 downregulates TWIST1 by recruiting HDAC1 to the TWIST1 promoter, which inhibits pancreatic cancer metastasis [56]. SOX2 recruits NuRD and cooperatively represses mTOR, inducing autophagy and subsequent reprogramming to pluripotency [57]. These intricate and complex interactions collectively contribute to the transcriptional regulation of the SOX family in both physiological and tumorigenic contexts.

### Epigenetic regulation of SOX TFs

Apart from PTM, SOX proteins are also regulated at epigenetic levels, which mainly involves methylation and non-coding RNAs (ncRNAs). DNA methylation typically leads to gene silencing and repression, which primarily occurs in SOX with a suppressor role in cancer. METTL14 raises YTHDF2 to m6A-modified sites of SOX4 mRNA and promotes SOX4 degradation [58]. The methylation of SOX11 promoter leads to SOX11 silencing in hematopoietic malignancies [59]. In lung cancers, loss of SOX30 frequently occurs due to its methylation [60]. The methylation of SOX17 promoter contributes to its epigenetic silencing in papillary thyroid carcinoma [61].

NcRNAs mainly include miRNAs and lncRNAs. LncRNAs can competitively bind to the target mRNA of miRNAs, or act as a molecular sponge to relieve the inhibition of miRNAs on target mRNA and promote the expression of target genes [62]. MiR-183-5p downregulates MUC15, inducing SOX2 expression via the c-MET/PI3K/AKT axis [63], while miR-653-5p upregulates SOX30 in prostate cancer [64]. LncRNA PCAT1 upregulates SOX2 in non-small cell lung cancer (NSCLC) [65]. In most cases, they collectively regulate the expression of SOX TFs. In cholangiocarcinoma cells, YY1 upregulates lncRNA DLEU1, which competitively binds to miR-149-5p, elevating Yes-associated protein 1 (YAP1) expression. YAP1 upregulates SOX2 via binding to TEAD on the promoter region of SOX2, ultimately promoting the proliferation of cholangiocarcinoma cells [66]. Besides, lncRNA PTV1 upregulates SOX2 expression by competitively bind to miR-136 [67]. LncRNA SNHG47 upregulates SOX4 by sponging miR-338-3p, promoting the proliferation of ESCC cells [68]. Additionally, SOX5 expression is upregulated by lncRNA SOX2OT via miR-194-5p, ultimately promoting colorectal cancer (CRC) invasion [69]. Moreover, SOX9 upregulates lncRNA FARSA-AS1 expression by binding to its promoter, while FARSA-AS1 upregulates SOX9 by impeding miR-18b-5p, forming a feedback loop to promote CRC metastasis [70]. Similarly, miR-424-5p sponged by LncRNA HCG18, miR-361-3p sponged by lncRNA PVT1 and miR-296-5p sponged by lncRNA PRR34-AS1 promote SOX9 expression in cholangiocarcinoma [71], NSCLC [72] and HCC [73] respectively. Altogether, methylation and ncRNAs epigenetically silence tumor-suppressor role of SOX proteins in cancers, while enhancing the expression of oncogenic SOX proteins, thereby facilitating tumor initiation and progression.

## SOX TFs in cancer

**Fig. 3:**
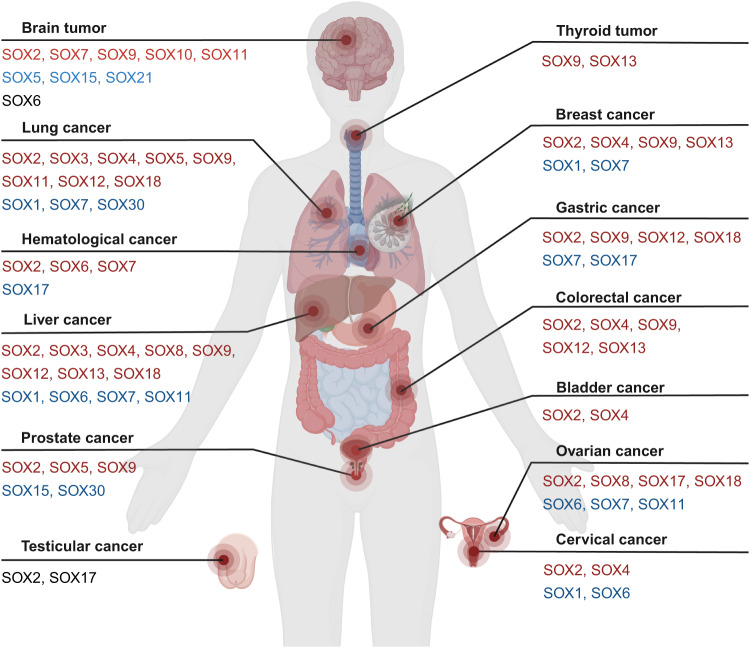
Involvement of human SOX TFs in cancer. The participation of SOX TFs in various cancers has been disclosed, including brain tumor [103, 105, 107, 113, 118, 125, 127, 131, 154, 155, 176, 177, 183, 184, 188, 194], thyroid cancer [98], lung cancer [41, 65, 73, 104, 135, 136, 151, 164, 193], breast cancer [121, 145, 146, 153, 158, 171, 181], hematologic cancer [123, 178], liver cancer [82, 84, 87, 89, 93, 95, 108, 111, 115, 132, 152], gastric cancer [91, 92, 112, 137, 138, 160, 190], colorectal cancer [58, 69, 70, 77, 85, 96, 110, 124, 149, 189], ovarian cancer [100, 120, 169, 173], bladder cancer [201], prostate cancer [64, 81, 90, 116, 117, 130, 163], testicular cancer [110, 195] and cervical cancer [114, 134]. The red fonts stand for upregulation, blue indicates downregulation and black for either upregulation or downregulation.

Cancer is characterized by eight core hallmarks, including the ability to sustain proliferative signaling, resist growth suppressors and cell death, enable replicative immortality, access vasculature, activate invasion and metastasis, reprogram cellular metabolism, and avoid immune destruction [74]. The SOX family influences these cancer characteristics through various mechanisms, encompassing promoting cancer invasion and metastasis, influencing proliferation and apoptosis, aiding in stemness, facilitating EMT, and modulating tumor immunity. Therefore, the disturbance of the SOX family runs through the occurrence and development of many tumors (Fig. 3).

### SOX family acts as an oncogene

**Fig. 4:**
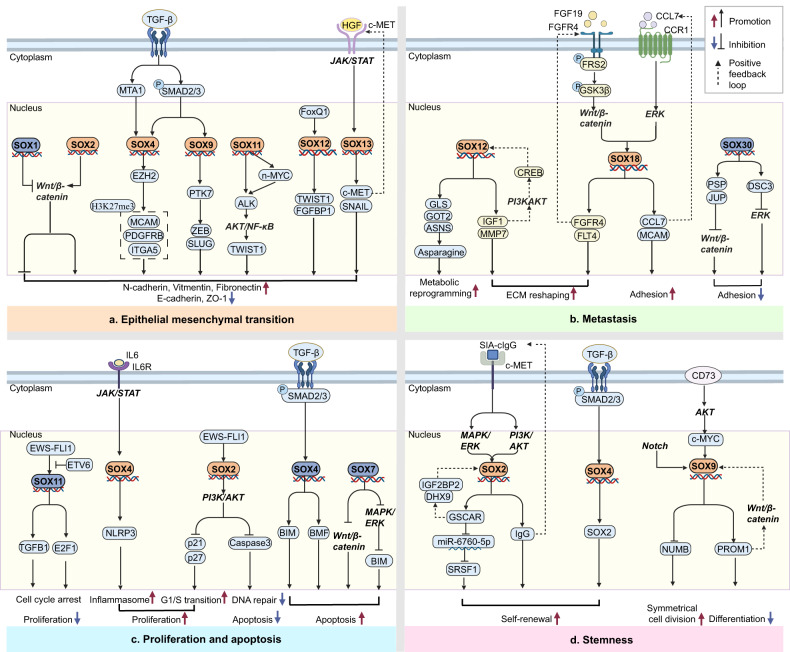
SOX TFs acts as the hub of signaling pathways in the tumorigenesis. SOX TFs in critical positions of the signaling pathway that mediates EMT [76, 82, 85, 87, 132, 78–80, 86], invasion and metastasis [91–93, 96, 135], proliferation and apoptosis [59, 97, 99, 123, 124, 128], stemness of cancer [104, 105, 107, 108, 110, 111].

Numerous members of the SOX family significantly contribute to the initiation and progression of tumors. They primarily promote EMT, invasion and metastasis, proliferation and apoptosis, and stemness of tumors. In some cases, they also relate to treatment insensitivity and poor prognosis (Fig. 4).

#### EMT

EMT refers to the process by which epithelial cells acquire interstitial features under certain conditions [75]. Hypoxia and cytokines secreted by stromal cells significantly induce EMT and promote tumor invasion for cells at the margins of many solid tumors [40]. Transcription factors are crucial in controlling multiple cellular events like proliferation, survival, differentiation, adhesion, and migration during the EMT [75]. So far, several transcription factors, including SNAIL, TWIST1, ZEB, and SLUG, are extensively involved in the EMT process. SOX TFs serve as the hub of these signaling pathways and mediate the EMT of tumors.

Wnt/β-catenin signaling pathway mediates EMT of tumors. Through activating the Wnt/β-catenin pathway, SOX2 induces EMT in breast cancer and prostate cancer [76]. Recent studies have demonstrated that DVL3 upregulates SOX2 expression by activating the Wnt/β-catenin/c-MYC pathway, thus promoting and maintaining EMT in CRC [77]. Besides, SOX family is a crucial mediator of the TGF-β-induced EMT in cancer. SOX4 is upregulated by TGF-β-induced-MTA1 and contributes to EMT via inducing expression of EZH2, which mediates H3K27me3 epigenetic imprint of Mcam, Pdgfrb, Itga5 [78, 79]. Aside from being a direct TGF-β target, SOX4 also performs as an effector of TGF-β signaling via interacting with SMAD3 in a phosphorylation-independent manner [80]. The upregulation of SOX5 by the TGF-β/SMAD3 signaling pathway activates TWIST1, ultimately contributing to EMT in prostate cancer [81]. The upregulation of SOX9 via the TGF-β/SMAD2/3 pathway induces the expression of PTK7, which subsequently upregulates ZEB1 and SLUG, leading to EMT in hepatic cell carcinoma (HCC) [82]. Moreover, SOX TFs induces EMT through directly stimulating the expression of SNAIL or TWIST. SOX3 induces EMT in osteosarcoma cells by transcriptionally activating SNAIL1 [83]. In HCC, ELMO1-induced upregulation of SOX10 activates the PI3K/AKT signaling pathway. Subsequently, this pathway enhances the expression of SNAIL, which ultimately induces EMT [84]. HGF upregulates SOX13 through the JAK/STAT pathway. Consequently, the upregulation of SOX13 promotes the EMT of CRC by transactivating SNAIL and c-MET [85]. SOX11 activates ALK directly or via upregulation of n-MYC, which activates the downstream transduction cascades containing AKT, NF-κB and TWIST1, leading to EMT in uterine carcinosarcoma [86]. FoxQ1 upregulates SOX12 expression, which transactivates the expression of TWIST1 and FGFBP1, promoting EMT in HCC [87].

#### Invasion and metastasis

Tumor invasion and metastasis are the major causes of cancer progression and treatment failure. Therefore, studying and understanding the mechanisms behind tumor invasion and metastasis are crucial for cancer treatment and prognosis. Through remodeling the metabolism, extracellular matrix and tumor microenvironment, SOX TFs contribute to the invasion and metastasis of tumors.

ECM is a non-cellular component of the extracellular matrix in tissues, containing various proteins, polysaccharides, and other molecules, providing structural support and biological signal transduction for cells [88]. By regulating ECM composition and structure, SOX promotes tumor metastasis. SOX9 promotes the secretion of activin B from HCC cells via upregulating INHBB. Activin B promotes the activation of surrounding hepatic stellate cells, which promotes the deposition of extracellular matrix, contributing to the metastasis of HCC [89]. Zbtb7a, a member of POK transcription factors, interacts with SOX9 to antagonize its transcriptional activity on key target genes. In advanced prostate cancer, the genetic loss of Zbtb7a activates SOX9-targeted genes such as H19 and MIA, ultimately reducing the expression of integrin to promote cancer metastasis [90]. In GC, SOX12 promotes tumor extracellular matrix (ECM) reshaping by upregulating matrix metalloproteinase (MMP) 7 and IGF1. In turn, IGF1 induces SOX12 expression through IGF1/CREB/SOX12 pathway, forming a feedback loop to promote GC metastasis [91]. The CCL7-CCR1 axis upregulates SOX18 via the ERK/ELK1 pathway. In turn, SOX18 induces the expression of MCAM and CCL7, forming a feedback loop to promote GC adhesion and metastasis [92]. In addition, Chen et al. discovered that in HCC, FGFR4 and its ligand FGF19 transactivate the SOX18 promoter directly by activating the p-FRS2/p-GSK3β/Wnt/β-catenin pathway. In turn, SOX18 directly increases the expression of FGFR4 and FTL4, forming a feedback loop to promote the ECM reshaping and metastasis of HCC [93].

Besides ECM, SOX TFs also mediate tumor metastasis via remodeling metabolism and microenvironment. SOX2 upregulates IL6 by inducing the expression of FOSL2. IL6 activates JAK/STAT signaling, sustaining inflammation to promote tumor metastasis [94]. SOX4 upregulates CXCL12 which promotes neovascularization of microenvironment via activating CXCL12/CXCR4/CXCR7 axis, ultimately catalyzing HCC metastasis [95]. Moreover, SOX12 transactivates GLS, GOT2 and ASNS, which promotes metabolic reprogramming by inducing asparagine synthesis, ultimately promoting CRC metastasis [96].

#### Proliferation and apoptosis

Evading growth suppressors and resisting cell death are two core hallmarks of cancer [74]. Through promoting G1/S transition, angiogenesis and activating inflammasome, SOX TFs promotes cancer proliferation. In Ewing sarcoma, SOX2 upregulated by EWS-FLI1 activates PI3K/AKT signaling. The activated AKT then promotes G1/S transition via inhibiting p21 and p27 proteins. Simultaneously, the activated AKT promotes poly (ADP-ribose) polymerase (PARP)-induced DNA repair through impeding caspase3, which suppresses cell apoptosis. Altogether, SOX2 promotes Ewing sarcoma growth [97]. SOX13 upregulates TRIM11, which inhibits the degradation of YAP, ultimately promoting the G1/S transition of anaplastic thyroid cancer cells [98]. In OSCC, SOX4 is upregulated by IL-6 through the JAK/STAT pathway. SOX4 induces the expression of NLRP3, promoting OSCC proliferation via activating inflammasome [99]. Moreover, The interaction between PAX8 and SOX17 promotes vascular endothelial growth factor (VEGFR) expression by downregulating SERPINE1, ultimately leading to angiogenesis in ovarian cancer (OC) [100].

#### Stemness

Cancer stem cells (CSCs) refer to a group of stem cells with abnormal differentiation and uncontrollable division [101]. Several signaling pathways, including Wnt/β-catenin, NF-κB, Notch, Hedgehog, PI3K and JAK/STAT, are vital in maintaining CSCs [101]. By acting as the upstream or downstream in these signaling pathways, the SOX family contributes to stemness of cancer cells via regulating the self-renewal ability, division and differentiation.

SOX2 is considered one of the four key transcription factors that mark the pluripotency along with KLF4, OCT3/4, KLF5 and MYC [102]. Hypoxia-inducible factor (HIF) induces the expression of both SOX2 and KLF4 in glioblastoma (GBM), which in turn upregulate CD133/15, ultimately promoting stemness expression [103]. In lung cancer, SIA-cIgG binds to c-Met, upregulating SOX2/OCT4 through the PI3K/AKT/mTOR and RAS/RAF/MAPK/ERK signaling pathways. In turn, SOX2 induces the expression of IgG and promotes its translocation to the cytoplasm, forming a feedback loop. Altogether, SOX2 promotes the self-renewal and cancer cell stemness [104]. Moreover, SOX2 upregulates the expression of lncRNA GSCAR, which competes with miR-6760-5p to upregulate SRSF1 expression, thereby maintaining glioma stem cell (GSC) self-renewal ability. Additionally, GSCAR enhances the interaction between IGF2BP2 and DHX9, promoting SOX2 stabilization and forming a positive feedback loop [105].

Induced by cisplatin, CLU triggers mitochondrial fission by activating DNM1L through AKT-mediated phosphorylation. CLU promotes the mitophagic degradation of MSX2 and induces the expression of SOX2, thereby enhancing stemness in oral cancer [106]. Han et al. discovered that SOX4 upregulated by TGF-β-SMAD2/3 signaling increases the self-renewal ability of glioma stem cells via inducing SOX2 expression, indicating a crucial role of SOX2 and SOX4 in stemness maintenance [107]. SOX9 is a critical regulator of cell stemness. Liu et al. discovered that SOX9 is upregulated via Notch signaling, which enhances cancer stem cells’ self-renewal and symmetrical cell division (SCD) of liver CSCs via downregulating Numb expression [108]. Song et al. reported that YAP1 induces SOX9 expression via interacting with TEAD, a conserved binding site in the SOX9 promoter. This upregulation of SOX9 increases SCD and maintains stemness [109]. In CRC, SOX9 directly activates PROM1 via a Wnt/β-catenin-responsive intronic enhancer. Additionally, PROM1 also upregulates SOX9 via stabilizing β-catenin. This interaction creates a positive feedback loop between SOX9 and PROM1, impeding differentiation and enhancing the stem cell properties of CRC [110]. CD73 upregulates SOX9 through the AKT/c-MYC signaling pathway and impedes its ubiquitination and subsequent degradation via inhibiting GSK3β, thus promoting dedifferentiation and maintaining the stemness of HCC [111].Recently, Chen et al. established a hereditary GC model expressing both SOX2 and SOX9, with distinctive cell populations and metastasis in the liver and peritoneum. They discovered that these metastatic cells originated from SOX9+ progenitor cells in the stomach, and SOX9 plays an indispensable role in the asymmetric division of GC stem cells [112].

#### Therapy resistance

The development of drug resistance in cancer treatment remains a significant obstacle to successful clinical outcomes. Therefore, exploring drug resistance mechanisms is paramount to enhancing therapeutic effect and patient prognosis.

The disturbance of ATP-binding cassette subfamily G member (ABCG) is implicated in SOX-induced drug resistance. In CD133-positive glioma stem cells (GSCs), the miR-145/OCT4/SOX2 axis confers radio-chemo resistance, while inhibition of this axis increases the sensitivity to temozolomide (TMZ) and radiation, following a decreased expression of ABCG2, MDR1 and Bcl2 [113]. SOX4 increases the expression of ABCG2 in cervical cancer and subsequently promotes resistance to cisplatin [114]. SOX9 upregulates ABCG2 via activating the AKT pathway, which confers sorafenib resistance of HCC [115].

Besides ABCG, multiple mechanisms like aberrant signaling pathways, including NF-κB, Wnt/β-catenin, AKT and mTOR, also participate in SOX-induced drug resistance. In prostate cancer, SOX2 dysregulates the cell cycle via upregulating WEE1 and CDK1, resulting in the insensitivity of prostate cancer to NHRSI [116]. Gao et al. discovered that in NSCLC, SOX2 suppresses radioimmune responses via activating the cGAS/STING signaling pathway [65]. The expression of SOX2 is inhibited by TP53 through the upregulation of miR-34, whereas RB1 represses SOX2 expression via directly binding to the E2F binding sites of the SOX2 promoter. The deficiency of TP53 and RB1 in prostate cancer promotes the expression of SOX2, contributing to antiandrogen resistance. Consequently, this leads to the transdifferentiation of treatment-sensitive adenocarcinoma cells into derivatives that resemble neuroendocrine cell states [117]. Furthermore, SOX4 promotes TMZ resistance in GBM by interacting with EZH2 and coactivating METTL3, resulting in transcriptional plasticity of tumor cells [118]. TEAD inhibitor induces SOX4 expression via upregulation of VGLL3, promoting cancer cell survival and resistance to TEAD inhibitors via activating the PI3K/AKT signaling pathway [119]. In OC, SOX8 phosphorylated by Aurora-A promotes glucose metabolism and inhibits cell senescence via upregulating FoxK1, ultimately promoting resistance to cisplatin [120]. By upregulating HDAC5, c-MYC promotes the deacetylation of SOX9, which subsequently leads to its nuclear translocation, contributing to tamoxifen resistance in breast cancer [121].

### SOX family acts as a suppressor

Despite most research focusing on the oncogenic characteristics of SOX TFs, they also exhibit anticancer effects in specific types of tumors. SOX TFs act as double-edged swords, exerting divergent effects in different tumor types via distinct signaling pathways. A comprehensive understanding of the multifaceted roles of SOX TFs in tumors is of great significance for elucidating the mechanisms of tumor development and informing therapeutic approaches.

#### Proliferation and apoptosis

Uncontrolled cell proliferation and apoptosis are key features in tumor development. Certain members of the SOX protein family can impede cell proliferation and promote apoptosis, thus inhibiting tumor growth and improving patient prognosis. TGF-β signaling is also a dual mediator of tumor proliferation and metastasis. During TGF-β/SMAD-induced EMT, the upregulation of SOX4 by TGF-β activates the pro-apoptotic proteins BIM and BMF, ultimately leading to apoptosis in pancreatic cancer. Notably, this shift of SOX4’s function from pro-tumor to pro-apoptosis is triggered by the suppression of KLF5, which is downregulated by TGF-β-induced SNAIL [122]. Overexpression of SOX11 inhibits proliferation in hematopoietic malignancies, with the activation of the TGF-β signaling pathway and reduction of E2F1, while the methylation of SOX11 promoter leads to SOX11 silencing and the proliferation of tumors [59]. Besides TGF-β, Wnt/β-catenin is a typical pro-tumor signaling. Through antagonizing the Wnt/β-catenin signaling pathway, SOX7 inhibits the proliferation of acute myeloid leukemia (AML) and CRC [123, 124]. Additionally, through downregulating Wnt/β-catenin signaling, SOX15 inhibits cell proliferation of glioma cells [125]. P53 is a crucial tumor suppressor gene that regulates cell growth, DNA repair, and apoptosis. In HCC, SOX6 inhibits NPM1 via repressing c-MYC, which subsequently stabilizes and activates p53 via promoting the formation of the p14ARF/HDM2/p53 complex, ultimately inhibiting HCC proliferation [126]. In primary human GBM cells, SOX5, SOX6, and SOX21 induce apoptosis by upregulating CDK inhibitors and downregulating p53 protein turnover [127]. In addition to these classical signaling pathways and interacting proteins, there are also other mechanisms involved in cancer inhibition ability of SOX. In lung cancer, SOX7 induces apoptosis through the MAPK/ERK/BIM pathway [128]. In Ewing sarcoma, ETV6 competes with EWS-FLI1 to bind SOX11. Inactivation of ETV6 enhances the interaction between EWS-FLI1 and SOX11, leading to increased expression of SOX11. This, in turn, causes cell cycle arrest and inhibits proliferation [129]. In prostate cancer, SOX15 functions as a tumor suppressor by upregulating AOC1, which reduces the proliferation and migration of prostate cancer through the activation of reactive oxygen species and ferroptosis [130]. Moreover, the overexpression of SOX21 forms a complex with SOX2, altering the balance and composition of SOX2 and SOX21, consequently inducing apoptosis in glioma cells [131].

#### Invasion and metastasis

Invasion and metastasis contribute to the poor prognosis and are responsible for the fatal outcome in cancer patients. Some members of the SOX family can inhibit cancer invasion and metastasis, elucidating the complex dual effects of SOX TFs in cancer. Specifically, through inhibiting the Wnt/β-catenin signaling pathway, SOX1 impedes EMT and subsequent metastasis in HCC [132], nasopharyngeal carcinoma [133], and cervical cancers [134]. Moreover, in lung adenocarcinoma, SOX30 downregulates Wnt/β-catenin and ERK signaling by promoting the transcriptional activation of DSP, JUP and DSC3, suppressing cancer adhesion and metastasis [135]. Similarly, SOX30 suppresses metastasis of lung cancer [136] and prostate cancer [136] via inhibiting Wnt/β-catenin signaling. Besides Wnt/β-catenin signaling, phosphatase and tensin homolog (PTEN) is another mediator of SOX’s suppressing role. Furthermore, SOX2 upregulates PTEN, which in turn dephosphorylates AKT, thereby suppressing invasion in GC [137]. The lncRNA BC002811 disrupts the interaction between SOX2 and PTEN, consequently suppressing PTEN transcription. The downregulation of PTEN ultimately promotes GC metastasis via upregulating MMP2 and MMP9 [138].

## SOX TFs in tumor immune microenvironment

**Fig. 5:**
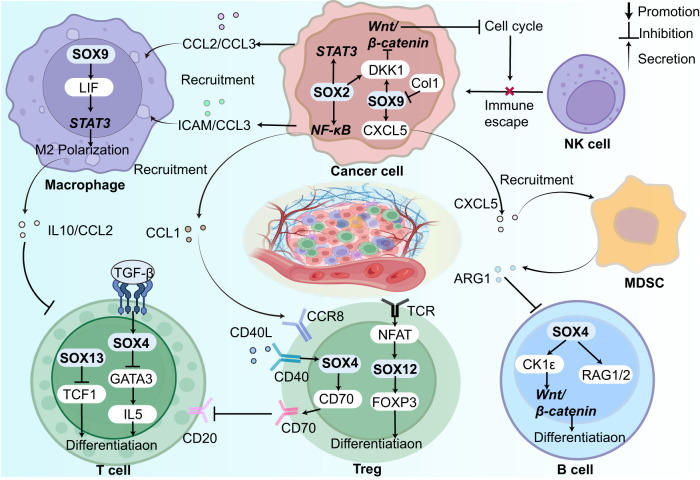
SOX TFs as multifaceted players in immune microenvironment. Extensive involvement of SOX factors in various kinds of immune cells, including TAMs [140, 145], Tregs [143, 153, 156], CD8 + T cell [140, 141, 160], B cell [144], NK cells [165] and MDSCs [167], renders them as crucial regulators and promising targets for immunotherapy.

The immune system monitors the internal environment, identifying and eliminating abnormal cells primarily through T cells, B cells, and natural killer (NK) cells. However, a suppressive TIME consisting of tumor-associated macrophages (TAMs), regulatory T cells (Tregs), and myeloid-derived suppressor cells (MDSCs) facilitates immune evasion and tumor cell survival. These cells interact via complex communication networks using secreted cytokines, chemokines, growth factors, and ECM proteins, contributing to a stable immune microenvironment [139]. SOX TFs regulate the differentiation, recruitment, and activation of these immune cells, thus maintaining the homeostasis of the immune microenvironment. Dysregulation of immune reactions, either excessive or weak, can lead to the development of diseases such as autoimmune diseases and cancer. In the context of cancer, the SOX family typically establishes a suppressive TIME by recruiting suppressive immune cells, enhancing the secretion of immune inhibitory molecules and suppressive cytokines, which confers resistance to immunotherapy in many patients (Fig. 5).

### SOX TFs inside immune cells

SOX TFs participate in the differentiation and maturation of multiple immune cells in both physiological and pathological contexts. Inside TAMs, Fan et al. demonstrated that SOX9-regulated leukemia inhibitory factor (LIF) mediates macrophage repolarization to an M2 state via activating STAT signaling. This repolarization process induces the secretion of immunosuppressive factors, including IL10 and CCL2, by TAMs, ultimately leading to immune suppression of T cells. In airway inflammation, SOX4 is upregulated by TGF-β and inhibits TH2 differentiation by downregulating GATA3-induced IL5 [140]. Additionally, SOX13 acts as a switch of αβ and γδ T cell differentiation via antagonizing TCF1 [141]. CD39 is expressed on some surfaces of human thymus-derived Tregs, which regulates the immune suppressive capacity of Tregs. The overexpression of CD39 in Tregs maintains its status via autocrine/paracrine TGF-β signaling, which induces CD39 expression via upregulating SOX4 [142]. Moreover, in adoptive transfer colitis, SOX12 is upregulated by T cell receptor (TCR)-mediated NFAT activation in CD4 + T cells. SOX12 induces Foxp3 expression via directly binding to its promoter, subsequently inducing the differentiation of Tregs in the periphery [143]. However, the relationship between SOX12 and Tregs in cancer remains unknown. Physiologically, SOX4 induces the differentiation of early B cells by inhibiting Wnt/β-catenin through upregulating CK1ε. Simultaneously, SOX4 also upregulates RAG1/2, thereby allowing for VDJ gene rearrangements at the Igh locus [144].

### SOX TFs in the interaction of cancer cells and immune cells

#### SOX TFs mediate interactions between cancer cells and TAMs/TANs

M2 TAMs and tumor-associated neutrophils (TANs) secrete many cytokines that contribute to a suppressive TIME for tumor survival. In breast cancer, the overexpression of SOX2 upregulates CCL3 and ICAM1 via activating NF-κB. Simultaneously, SOX2 induces CCL2 and CCL3 via activating STAT3 signaling pathway. These changes ultimately result in the recruitment of TAMs in the TME [145]. The secretion of CXCL1 by TAMs promotes the upregulation of SOX4 by activating NF-κB signaling pathway, ultimately promoting breast cancer metastasis [146]. In GSCs, the co-expression of SOX2 and OCT upregulates BRD4, which subsequently promotes the secretion of cytokines and chemokines, such as SPP1, CXCL5, and IL8, ultimately leading to the recruitment and polarization of M2 TAMs [147]. SOX2 is upregulated in head and neck squamous cell carcinoma (HNSCC) due to the enhancing effect of TAMs on the availability of HA, which binds to CD44 receptors on the surface of cancer cells. Through activating PI3K/4EBP1 axis, CD44 upregulates SOX2, thereby promoting the CSC properties of the cancer cells [148]. In CRC, the expression of SOX4 can be induced by TGF-β, which is secreted by TAMs under hypoxia stimulation. As a result of this upregulation, SOX4 increased the expression of TMEM2 and concomitantly inhibited endoplasmic reticulum stress (ERS) in CRC cells [149]. PDGF-BB, which is secreted by tumor cells, facilitates the expression of IL-33 through the upregulation of SOX7. This molecular cascade subsequently promotes cancer metastasis by recruiting TAMs [150]. TANs recruitment is driven by the upregulation of CXCL5 mediated by SOX2 and the loss of NKX2-1 [151].

#### SOX TFs mediate interactions between cancer cells and Tregs

Tregs are a subset of CD4 + CD25 + T cells that exert a significant impact on maintaining the body’s immune homeostasis. However, in the tumor microenvironment, the suppressive effect of Tregs inhibits the anti-tumor response of T cells and weakens the body’s immune response against tumors, thereby promoting tumor growth [152]. SOX2 reduces the binding of H3K27Me on the promoter region of CCL1, inducing CCL1 expression. Simultaneously, SOX2 upregulates CCL1 via activating NF-κB signaling pathway, ultimately recruiting Tregs to the TME, which maintains the CSCs of breast cancer [153]. In high-grade glioma, deletion of SOX7 reduced CCL17, CCL22, and CCL5, which subsequently decreased Treg cells, while deletion of SOX17 increased CCL17 concentration and recruitment of Treg cells [154]. Xiao et al. analyzed data from TCGA and CCGA and identified an association between SOX10 overexpression in glioma and DNA replication, mismatch pair and regulation of negative regulatory T-cell differentiation [155]. Balsas et al. found that SOX11 expression in mantle cell lymphoma (MCL) is correlated with CD70 overexpression, increased Tregs infiltration and T-cell activation, contributing to the invasion of MCL [156].

#### SOX TFs mediate interactions between cancer cells and responsive T/B cells

Responsive T cells and responsive B cells are the primary effector cells in the human body that carry out immune clearance, kill pathogenic cells, and maintain the microenvironment. CD8 + T cell is the main cytotoxic T cell in responsive T cells, which can eliminate tumor cells through the production of cytokines, activation of memory T cells, or direct cytotoxic effects. Responsive B cells mainly produce specific antibodies to help eliminate tumor cells or inhibit their growth. In HNSCC, Tan et al. found that SOX2 inhibits the infiltration of CD8 + T cells via inhibiting STING-type I interferon (IFN) signaling and facilitating autophagy-dependent degradation of STING, which contributes to the immune escape of tumors [157]. In triple-negative breast cancer, integrin αvβ6 on the surface of epithelial cancer cells activates TGF-β to induce SOX4 expression, promoting the immune escape via suppressing cytotoxic CD8 T cells [158]. Chimeric antigen receptor (CAR) T cell therapy remains ineffective in solid tumor environment for the exhaustion in TME. Good et al. found that this dysfunction of CAR T cells, including the transition of conventional CD8 + T-to-NK-like T cells, can be prevented by the downmodulation of ID3 and SOX4 efficiency [159]. SOX9 continuously suppresses CD8 + T cells in GAC patients through the upregulation of LIF or the recruitment of TAMs mentioned above, which is independent of its suppression of NK cells in PC patients [160]. As for B cells, few SOX family members have been found to be associated with B cells in TIME. In MCL, SOX11 blocks the B-cell differentiation program and activates the B-cell receptor (BCR) signaling pathway, leading to MCL’s oncogenesis and aggression [156].

#### SOX TFs mediate interactions between cancer cells and NK cells

NK cells are lymphocytes lacking a specific antigen receptor that can eliminate tumor and virus-infected cells without prior sensitization [161]. Through analyzing the TIMER 2.0 database, Qin et al. concluded that NK cells were negatively correlated with most SOX family genes, namely SOX4, SOX8, SOX11, SOX17 and SOX18 [162]. SOX9 activates NANOG, which represses ICAM1 expression by inhibiting histone acetyltransferase p300 recruitment to ICAM1 promoter. This leads to immune evasion of pancreatic cancer from NK cells and promotes pancreatic cancer recurrence [163]. Moreover, SOX2 and SOX9 have been found to confer the latency CSCs the ability to evade NK cell killing via inducing MHC1 markers of self, promoting metastatic relapse [164]. Malladi et al. discovered that Latency Competent Cancer (LCC) in lung and breast cancer, which expresses high levels of SOX2 and SOX9, undergoes autocrine Wnt/β-catenin inhibition through DKK1. This inhibition results in a slow-cycling state in LCC cells, leading to their escape from recognition by NK cells and eventually causing an enrichment of LCC [165].

#### SOX TFs mediate interactions between cancer cells and MDSC

Myeloid-derived suppressor cell (MDSC) is a group of immature immune cells derived from hematopoietic stem cell (HSCs) in the bone marrow, which inhibits immune responses to promote tumor growth and escape [166]. In pancreatic cancer, Chen et al. found that the upregulation of SOX9 occurs due to the deletion of Col1, leading to the elevated expression and secretion of CXCL5. Consequently, MDSCs are recruited to the tumor site, where they mediate the suppression of T and B cell function through the secretion of arginase, ultimately leading to the progression of pancreatic cancer [167].

### Association between the expression of SOX TFs and immune checkpoints in cancers

**Fig. 6:**
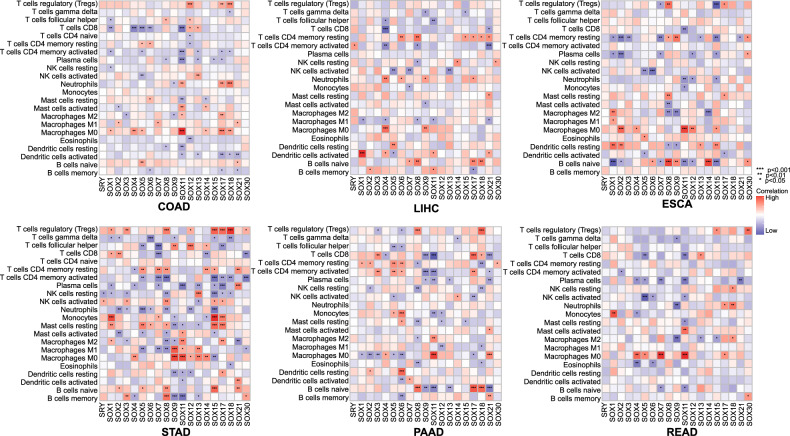
The association heatmap of SOX transcription factors expression in immune cells in digestive cancers. The association heatmap between SOX transcription factor expression and immune cells in digestive system malignancies. Red indicates positive correlation and blue indicates negative correlation. The color is darker, the correlation is stronger. The data was obtained from the TCGA public database. STAD stomach adenocarcinoma, LIHC Liver hepatocellular carcinoma, ESCA esophageal carcinoma, PAAD pancreatic adenocarcinoma, READ rectum adenocarcinoma, COAD colon adenocarcinoma.

The unresponsive TIME contributes to the ineffectiveness of many anticancer drugs. By manipulating immune checkpoints, tumor cells can regulate the activity of immune cells and facilitate immune evasion. The therapeutic effects of several anticancer drugs are greatly enhanced by combining them with immune checkpoint inhibitors (ICIs). An instance is the front-line treatment for liver cancer, where Lenvatinib and ICIs are combined [168]. More research is required to explore in-depth the underlying mechanisms and improve the therapy efficacy. Using TCGA data, we analyze the correlation between the SOX family and the infiltration of 22 immune cells in a panel of six common digestive tumors (Fig. 6). These heatmaps illustrate a clear correlation between the SOX family and the vast majority of immune cells in these tumors. In most studied tumors in the digestive system, SOX18 exhibits a strong correlation with Tregs, while SOX11 is highly correlated with TAMs and CD4 + /CD8 + T cells. These potential associations may inform future studies of tumor immunity.

**Fig. 7:**
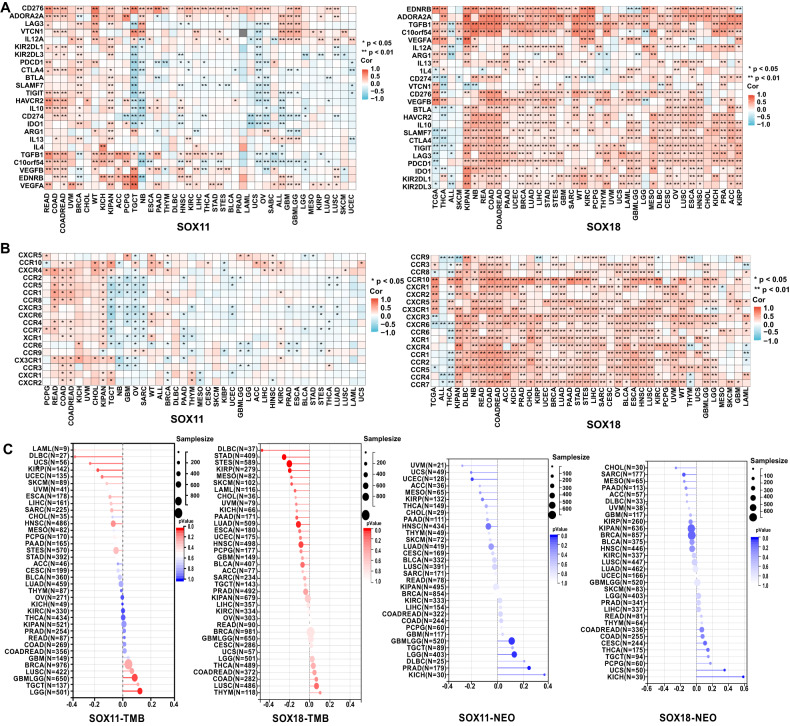
Pan-cancer-expression, immune checkpoints association, TMB and NEO of SOX TFs in tumors. Correlation between the expression of SOX TFs and that of immune-related genes in cancers. The pan-cancer dataset (TCGA TARGET GTEx, PANCAN, N = 19131, G = 60499) is downloaded from the UCSC database (https://xenabrowser.net/). After filtering out all normal samples and samples with zero expression levels, we performed log2(x + 1) transformation on each expression value and calculated the correlation between SOX family and the above indicators. We extracted the expression data of (**A**) immune checkpoint pathway genes (Inhibitory [24]); (**B**) Chemokine and their receptor genes (Chemokine [41], Receptor [18]) in each sample. Besides, the Simple Nucleotide Variation dataset is acquired from GDC (https://portal.gdc.cancer.gov/). The (**C**) tumor mutational burden (TMB) for each cancer is calculated using the R package maftools. Neoantigen data is obtained from previous studies [202]. Red indicates positive correlation and blue indicates negative correlation. The darker the color, the stronger the correlation. ACC adrenocortical carcinoma, BLCA bladder urothelial carcinoma, BRCA breast invasive carcinoma, CESC cervical squamous cell carcinoma and endocervical adenocarcinoma, CHOL cholangiocarcinoma, COAD colon adenocarcinoma, COADREAD colon adenocarcinoma/rectum adenocarcinoma, DLBC lymphoid neoplasm diffuse large B-cell lymphoma, ESCA esophageal carcinoma, FPPP FFPE Pilot Phase II, GBM glioblastoma multiforme, GBMLGG glioma, HNSC head and neck squamous cell carcinoma, KICH kidney chromophobe, KIPAN pan-kidney cohort (KICH + KIRC + KIRP), KIRC kidney renal clear cell carcinoma, KIRP kidney renal papillary cell carcinoma, LAML acute myeloid leukemia, LGG brain lower grade glioma, LIHC liver hepatocellular carcinoma, LUAD lung adenocarcinoma, LUSC lung squamous cell carcinoma, MESO mesothelioma, OV ovarian serous cystadenocarcinoma, PAAD pancreatic adenocarcinoma, PCPG pheochromocytoma and paraganglioma, PRAD prostate adenocarcinoma, READ rectum adenocarcinoma, SARC sarcoma, STAD stomach adenocarcinoma, SKCM skin cutaneous melanoma, STES stomach and esophageal carcinoma, TGCT testicular germ cell tumors, THCA thyroid carcinoma, THYM thymoma, UCEC uterine corpus endometrial carcinoma, UCS uterine carcinosarcoma, UVM uveal melanoma, OS osteosarcoma, ALL acute lymphoblastic leukemia, NB neuroblastoma, WT high-risk Wilms tumor.

Based on the findings above, we infer that SOX11 and SOX18 have an intimate connection with tumor immunity. Subsequently, we examined the co-expression of these two genes in immune checkpoints associated with cancer (Fig. 7A) and analyzed their correlation with chemokine and receptor (Fig. 7B), tumor mutation burden (TMB), and Neoantigen (NEO) (Fig. 7C). As shown in the figures, SOX18 exhibits a strong positive correlation with several immune checkpoints, including TNFRSF4, CX3CL1, ENTPD1, and SELP, as well as chemokines present in tumors such as EDNRB, ADORA2A, TGFB1, CCL2, CXCL12. Moreover, SOX11 and SOX18 are closely associated with tumor TMB and NEO, which is of significant importance in predicting immune efficacy and patient prognosis, providing new treatment strategies for personalized immunotherapy. These findings offer a new perspective for a deeper understanding of tumor immunology and hold promise for further opportunities in the research and treatment of tumor-associated antigens.

In summary, the SOX family significantly contributes to the tumor immune microenvironment by interacting with various components that jointly mediate immune evasion in tumors and serve as a potential prognosis biomarker in tumor immunotherapy.

## SOX and tumor treatment

Despite years of research and drug development, many patients are still deprived of suitable cancer treatment options. The roles and mechanisms of SOX TFs in tumors have garnered increasing attention, stimulating research into therapeutic strategies targeting SOX proteins. As transcription factors, the SOX family has long been considered undruggable due to the absence of binding sites for small molecule drugs that target these proteins [169]. Thus, the primary methods currently involve developing drugs that interfere with upstream and downstream of SOX TFs, designing peptides derived from SOX and stimulating immune responses (Table 2).

**Table 2:** SOX-targeted therapeutics.

Compounds	Target	Biomarker	Cancer	Mechanism	Reference
BAY-11-7082	CXCL1	SOX4	Breast cancer	Inhibits the EMT process via blocking NF-κB pathway	[146]
Rapamycin	mTOR	SOX2, SOX9	GBM	Inhibits the properties of GSCs	[177]
Gemcitabine	EGFR	SOX9	PaC	Inhibits the metastasis of PaC via inhibiting EGFR/FOXA2/SOX9 axis	[179]
Luoteolin	PI3K/AKT	SOX2	ESCC	Downregulates SOX2 through inhibition of the PI3K/AKT signaling pathway	[174]
Thiolutin	PSMD14	SOX2	HNSCC	Promotes chemosensitivity via inhibiting E2F1/AKT/SOX2 axis	[175]
P42	SOX2	SOX2	ESCC	Interacts with SOX2 via a constrained peptide expression cassette	[180]
ZF-ATFs	SOX2	SOX2	/	Binds to the promoter of SOX2 with a transcriptional repressor SKD domain.	[181]
Peptide	S9pep	/	Colon cancer	Mimics SOX9 tumor-suppressive properties and inhibit colon cancer cell growth	[182]
	S6pep	/	GBM	Induces SOX6 peptide-specific cytotoxic T lymphocytes, lyse GSCs derived from GBM.	[183]
	S11pep	/	Glioma	Generates SOX11-specific CD8 + T cells	[184]

*EGFR* epidermal growth factor receptor, *ESCC* esophageal squamous cell carcinoma, *GBM* glioblastoma, *GSC* glioma stem cell, *HNSCC* head and neck squamous cell carcinoma, *PaC* pancreatic cancer, *SOX* sex determining region Y (SRY)- HMG box.

### SOX TFs as biomarkers

Since SOX TFs have a tight connection with the development, drug resistance and prognosis of tumors, SOX TFs have been extensively studied as potential prognostic biomarkers. Although there are established biomarkers for stem cells in HCC, Ruzinova et al. conducted a comparison of the prognostic value of positive SOX9 immunohistochemistry with other markers, such as EpCAM and K19 in a large cohort of North American patients (*n* = 216). The findings indicate that SOX9 outperforms K19 and EpCAM as a stem cell marker in predicting the prognosis for HCC [170]. SOX10 is an immunochemical biomarker with moderate sensitivity and high specificity for the basal-like intrinsic subtype of breast cancer, which is independent of hormone receptor status [171]. SOX11, cooperated with CD23 and CD200, is considered the best-characterized biomarker in the leukemic variant MCL [172]. Moreover, with high sensitivity and specificity, SOX17 is overexpressed in most ovarian and endometrial cancers, making it a proper diagnostic biomarker [173].

### Targeting SOX TFs indirectly

SOX TFs are involved in various processes in tumorigenesis, such as EMT, stemness, proliferation and apoptosis, invasion and metastasis, as mentioned before. Targeting the upstream and downstream signaling pathways of SOX can inhibit these processes and, in turn, improve patient prognosis. For instance, Luoteolin, a kind of natural flavonoid, attenuates cancer cell stemness in paclitaxel-resistant esophageal cancer cells, which is achieved by downregulating SOX2 through inhibition of the PI3K/AKT signaling pathway [174]. In HNSCC, PSMD14 promotes chemoresistance via enforcing the E2F1/AKT/SOX2 axis. Therefore, Thiolutin, a PSMD14 inhibitor, robustly enhances chemosensitivity and improves patient prognosis [175]. Polydatin, a monocrystalline compound obtained from Polygonum cuspidatum, impedes GBM invasion via inhibiting multiple components of the epidermal growth factor receptor (EGFR)-AKT/ERK1/2/STAT3-SOX2/SNAIL signaling pathway [176]. Besides, cyclopamine, an inhibitor of the SHH pathway, combined with the mTOR inhibitor rapamycin, reduces the expression of SOX2 and SOX9 in GBM cell lines and subsequently inhibits the properties of GSCs [177]. In addition, using BAY-11-7082, a NF-κB inhibitor, hampers the CXCL-induced NF-κB/SOX4 interaction, which inhibits the metastasis of breast cancer [146]. Inhibition of the protein lysine methyltransferase G9A suppresses the self-renewal of chronic myeloid leukemia (CML) by upregulating SOX6 [178]. Moreover, gemcitabine, an EGFR inhibitor, reduces the metastasis of pancreatic cancer via inhibiting the EGFR/FOXA2/SOX9 axis [179].

### Targeting SOX TFs directly

Several methods are available to interfere with SOX TFs directly. Liu et al. designed a P42 peptide that can interact with SOX2 and inhibit its function without affecting its levels. The results showed that this peptide effectively inhibits tumor growth and metastasis without impacting normal esophageal epithelium [180]. Additionally, SOX2 is silenced by designing sequence-specific artificial transcription factors (ZF-ATFs) based on the ZF domain. The ZF-ATFs bind to the SOX2 promoter using a transcriptional SKD domain that represses their transcription [181]. With a minimum off-target effect and the easiness of delivery, ZF-ATFs would become a promising method for tumor treatment.

### Peptides derived from SOX TFs

Due to the suppressive role of SOX families in certain tumors, peptides that replicate their tumor-suppressing function are developed. Blache et al. synthesized a short SOX9 peptide located at the hinge between the HMG DNA-binding domain and the SOX E central conserved domain. This peptide mimics the tumor-suppressive properties of SOX9 and has been shown to inhibit the Wnt/β-catenin signaling pathway, impeding CRC growth [182]. Moreover, SOX TFs are promising tumor-associated antigens that only overexpressed in tumor cells. Peptides derived from SOX TFs are developed as antigen peptide epitopes to stimulate T cell response. Ueda et al. synthesized a SOX6 peptide that induces specific cytotoxic T lymphocytes capable of lysing GSCs derived from GBM [183]. Recently, a peptide derived from the amino acid sequence of SOX11 has been developed to treat GBM. This FMACSPVAL peptide demonstrates the highest efficiency in generating SOX11-specific CD8+ T cells, making it a promising epitope for T cell-based immunotherapy in glioma [184].

## Conclusion and prospects

Over the last three decades, the role of SOX transcription factors in various tumors has gradually been uncovered, including their involvement in metastasis, invasion, proliferation, apoptosis, EMT, stemness, and drug resistance. The Wnt/β-catenin, Notch, TGF-β, TWIST1, JAK/STAT, and NF-κB pathways are the main pathways that intimately interact with SOX TFs. However, most research has focused on a few members of the SOX family, such as SOX2, SOX4, and SOX9, while the characters of other SOX TFs in tumors require further research. Additionally, the research regarding SOX family and TIME is just getting started. Although we have identified some SOX family members closely associated with immunity, further experiments are needed to validate their roles and more detailed mechanisms in TIME. Moreover, despite TFs being undruggable, many drugs have been developed targeting these factors. However, relevant therapeutic drugs that have been put into clinical practice remain limited. Therefore, extensive research on SOX’s roles in tumors, particularly in TIME, has excellent prospects for advancing novel therapies.

## Data Availability

The datasets generated and/or analysed during the current study are available in the UCSC database (https://xenabrowser.net/) and GDC database (https://portal.gdc.cancer.gov/).
